# Microbiota of MR1 deficient mice confer resistance against *Clostridium difficile* infection

**DOI:** 10.1371/journal.pone.0223025

**Published:** 2019-09-27

**Authors:** Ashley D. Smith, Elissa D. Foss, Irma Zhang, Jessica L. Hastie, Nicole P. Giordano, Lusine Gasparyan, Lam Phuc VinhNguyen, Alyxandria M. Schubert, Deepika Prasad, Hannah L. McMichael, Jinchun Sun, Richard D. Beger, Vahan Simonyan, Siobhán C. Cowley, Paul E. Carlson

**Affiliations:** 1 Laboratory of Mucosal Pathogens and Cellular Immunology, Division of Bacterial Pathogens and Allergenic Products, Office of Vaccines Research and Review, Center for Biologics Evaluation and Research, United States Food and Drug Administration, Silver Spring, Maryland, United States of America; 2 High-performance Integrated Personal Environment, Center for Biologics Evaluation and Research, United States Food and Drug Administration, Silver Spring, Maryland, United States of America; 3 Division of Systems Biology, National Center for Toxicological Research, United States Food and Drug Administration, Jefferson, Arkansas, United States of America; University of Illinois at Urbana-Champaign, UNITED STATES

## Abstract

*Clostridium difficile* (*Cd*) infection (CDI) typically occurs after antibiotic usage perturbs the gut microbiota. Mucosa-associated invariant T cells (MAIT) are found in the gut and their development is dependent on Major histocompatibility complex-related protein 1 (MR1) and the host microbiome. Here we were interested in determining whether the absence of MR1 impacts resistance to CDI. To this end, wild-type (WT) and MR1^-/-^ mice were treated with antibiotics and then infected with *Cd* spores. Surprisingly, MR1^-/-^ mice exhibited resistance to *Cd* colonization. 16S rRNA gene sequencing of feces revealed inherent differences in microbial composition. This colonization resistance was transferred from MR1^-/-^ to WT mice via fecal microbiota transplantation, suggesting that MR1-dependent factors influence the microbiota, leading to CDI susceptibility.

## Introduction

Major histocompatibility complex-related protein 1 (MR1) is a highly conserved receptor that presents non-peptide antigens to specialized subsets of T-cells including mucosa-associated invariant T (MAIT) cells and other less characterized MR1-restricted T cells [1–4]. The specific metabolites presented by MR1 are derived from the riboflavin biosynthesis pathway, which mammals cannot produce; however, these metabolites are synthesized by many bacteria and fungi [5]. MAIT cells are dependent on MR1 and the host microbiota for their development and peripheral expansion; therefore, germ-free mice lack detectable MAIT cells [6]. In addition, MAIT cells are present in high proportions at mucosal sites, and differ from conventional T-cells in both function and antigen recognition [7]. Specifically, MAIT cells function as innate-like T-cells and can respond quickly by producing pro-inflammatory cytokines and secreting perforin/granzyme for direct cytotoxic effect [8]. Despite MAIT cells having a clear role in pulmonary infection including *Francisella tularensis*, *Mycobacterium tuberculosis*, and influenza virus [9–11], the contribution of MR1 to defense against gastrointestinal infections remains relatively unexplored. The observation that MAIT cells were activated and depleted from the peripheral blood of patients during acute cholera infection suggests that MAIT cells participate in immune responses to gastrointestinal infections [12]. Furthermore, MAIT cells were activated following *in vitro* stimulation with various intestinal pathogens and microbiota constituents, including *C*. *difficile*, *S*. *flexneri*, and *E*. *faecalis* [8, 13, 14].

*Clostridium difficile* (*Cd*) is a Gram-positive, spore-forming anaerobic bacterium that infects the gastrointestinal tract [15]. In the United States, there are ~500,000 cases of *Cd* infection (CDI) resulting in ~29,000 deaths annually [16]. Treatment with broad-spectrum antibiotics, the primary risk factor for development of CDI, perturbs the gut microbiota, leading to a loss of colonization resistance against *Cd*. CDI results in a range of symptoms including antibiotic-associated diarrhea, pseudomembranous colitis, toxic megacolon, and death. Although oral antibiotics are effective, CDI recurrence occurs in 20–35% of patients [17, 18]. Fecal microbiota transplantation (FMT), currently considered an effective treatment for recurrent CDI, introduces the microbiota from a healthy donor into a diseased recipient to restore colonization resistance [19, 20]. Considering *Cd* possesses the riboflavin biosynthetic pathway capable of synthesizing the metabolites presented by MR1, and a recent study showed that MAIT cells responded to PBMCs stimulated with fixed *Cd in vitro* [13], we were interested in determining whether the absence of MR1 increases susceptibility to CDI. Surprisingly, we found that MR1^-/-^ mice were not more susceptible to CDI, but instead were resistant to Cd colonization. Results from our study led us to conclude that the microbiota associated with the MR1^-/-^ mice differs from wild-type mice in that it is resistant to antibiotic disruption and therefore confers resistance against *Cd* colonization.

## Results

### *Clostridium difficile* does not colonize cefoperazone treated MR1^-/-^ mice

**Fig 1:**
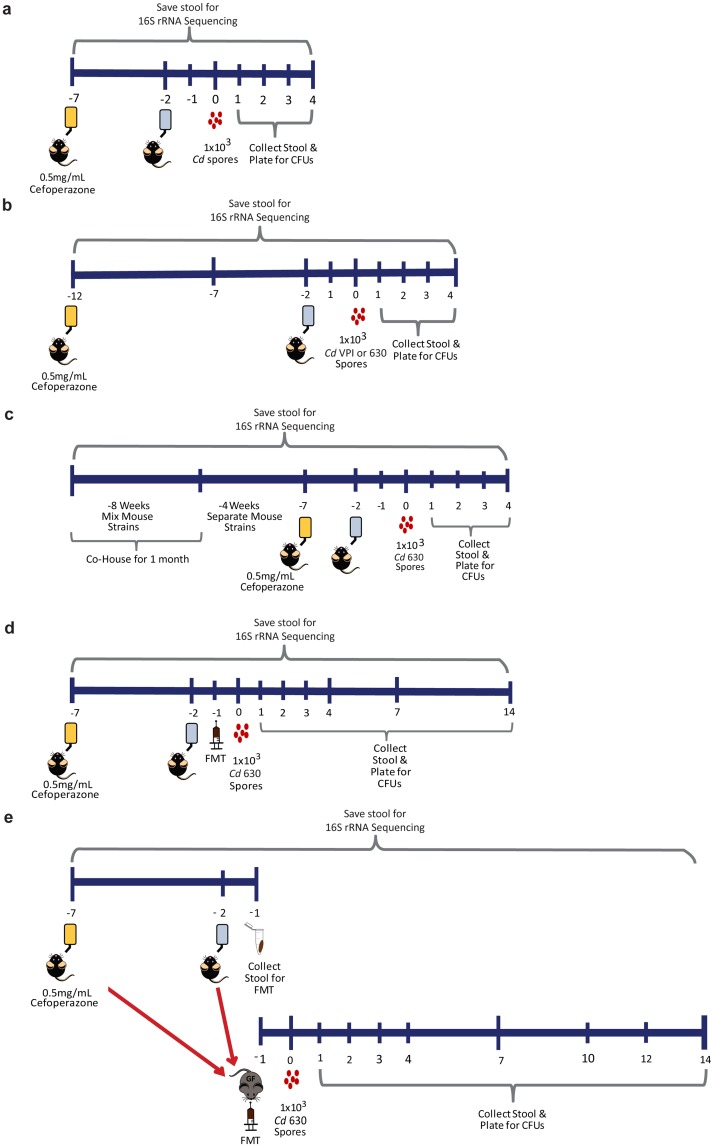
Experimental design for experiments performed in this study. (A) Standard model of CDI. Mice were given antibiotics in drinking water 5 days, normal water for two days rest, then inoculated with 1x10^3^ *Cd*630 spores. (B) Ten-day antibiotic treatment with inoculation of *Cd*630 or *Cd*VPI strains. (C) Co-housing strategy. (D) FMT of conventional SPF WT and MR1^-/-^ mice. (E) FMT of SPF antibiotic treated or untreated donor stool into WT germ-free recipients.

**Fig 2:**
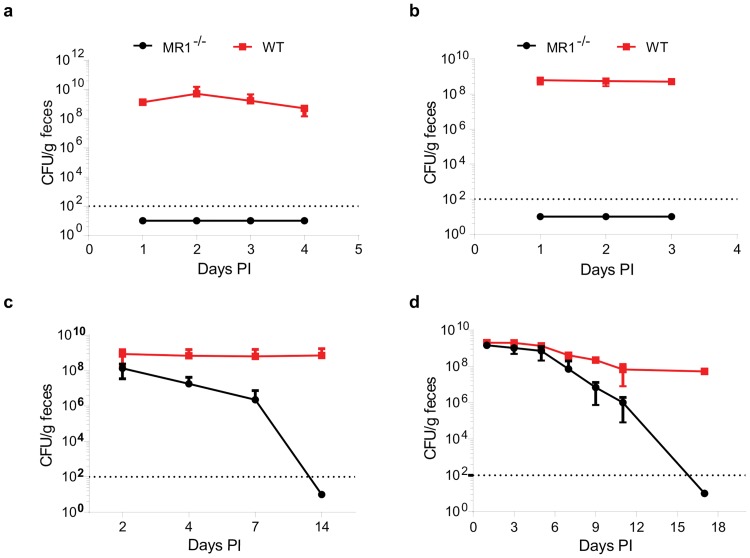
MR1^-/-^ mice exhibit resistance to *Cd* colonization in multiple infection models. (A) Cd colonization in WT and MR1^-/-^ mice in the standard cefoperazone model of CDI. WT n = 9, MR1^-/-^ n = 8. Data presented are mean ±SD. (B) Colonization levels were determined for WT and MR1^-/-^ mice pretreated with cefoperazone then inoculated with vegetative Cd cells and followed for three days PI. WT n = 5, MR1^-/-^ n = 6. Data presented are mean ±SD. (C) Colonization levels in WT and MR1^-/-^ mice for 14 days after streptomycin pretreatment and inoculation with Cd spores. n = 11 mice per group. Data presented are mean ± SD of colonization results from all mice across three independent experiments (n = 11 per group total). (D) Mice were pretreated with a cocktail of five antibiotics in the drinking water for five days followed by a switch to normal water and a single IP injection of clindamycin one day prior to inoculation with Cd spores. Cd colonization was followed for 17 days PI. n = 11 mice per group. Data presented are mean ±SD.

Since MAIT cells are found in high proportions in the gut, we hypothesized that mice lacking MR1, and therefore MAIT cells, would be more susceptible to infection with an intestinal pathogen such as *Cd*. Wild-type C57BL/6J and MR1^-/-^ mice were pretreated with cefoperazone for five days then given a two-day rest prior to infection with *Cd*630 spores (Fig 1A). Surprisingly, no *Cd* could be detected in the feces of MR1^-/-^ mice throughout the experiment (Fig 2A). To assess whether there was a difference in germinant availability in the MR1^-/-^ mice, we infected these mice with vegetative *Cd* following antibiotic treatment. Although WT mice were colonized, MR1^-/-^ mice remained resistant showing that this protection was not germination related (Fig 2B). Pre-treatment of mice with streptomycin (instead of cefoperazone) before inoculation also resulted in MR1^-/-^ mice exhibiting increased *Cd* colonization resistance (Fig 2C). Similar results were observed in a third model of CDI commonly used by many research groups [21–23]. This model uses pretreatment of mice with a 5-antibiotic cocktail in the drinking water followed by a single i.p. injection of clindamycin one day prior to inoculation with *C*. *difficile* spores by oral gavage [23]. Like the cefoperazone and streptomycin pretreatments, this model also showed increased colonization resistance and clearance in MR1^-/-^ mice (Fig 2D), indicating that resistance to persistent colonization is not limited to a specific antibiotic treatment. The alternative antibiotic pretreatment strategies were chosen as they have previously been shown to lead to *Cd* colonization [21, 24]. Each of these antibiotic pre-treatments is thought to allow for *Cd* colonization by reducing the overall diversity of the microbiota, which is normally protective against infection [21, 22, 25]. There is also evidence that this susceptibility is associated with changes in the bile acid content in these mice [25].

**Fig 3:**
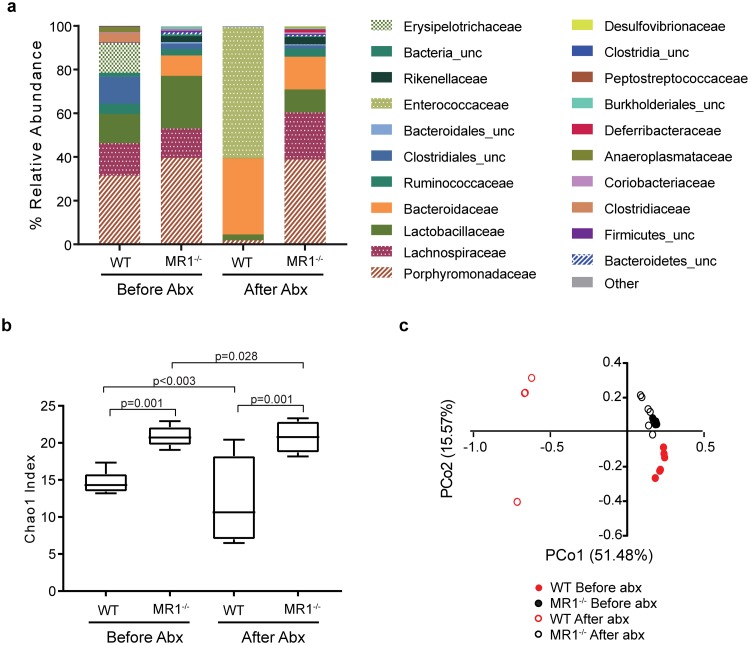
The gut microbiota composition of MR1^-/-^ mice contrasts that of WT mice before and after cefoperazone treatment. (A) 16S rRNA gene sequencing was performed on fecal samples from mice before and after antibiotic treatment. Data shown are mean % relative abundance at the family level. “_unc” denotes sequences unclassified at the family level; the lowest level identified is listed. Only taxa with ≥ 1% are presented. Data presented are mean ±SD; before antibiotics: WT n = 6, MR1-/- n = 6; after antibiotics: WT n = 5, MR1-/-n = 7. (B) Chao1 richness estimations of alpha diversity among all samples analyzed in panel A before and after antibiotic treatment. (C) Principle coordinate analysis (PCoA) of ThetaYC distances. Before antibiotics: WT n = 6, MR1-/- n = 6. After antibiotics: WT n = 5, MR1-/-n = 7.

**Fig 4:**
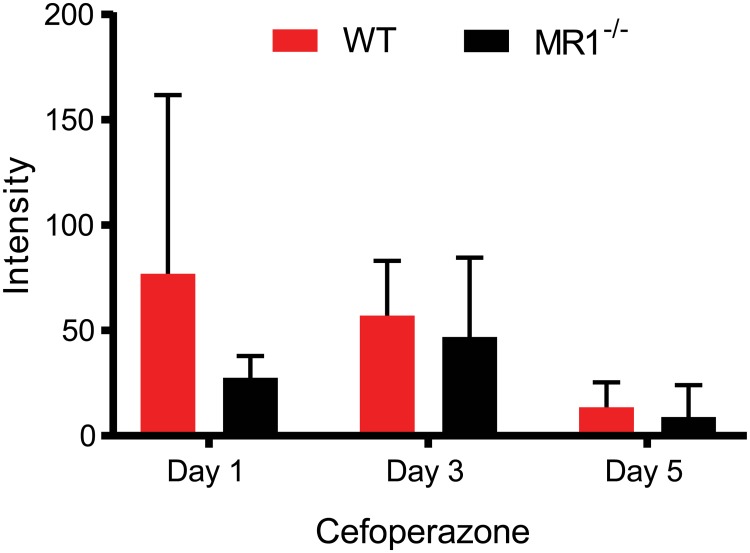
Cefoperazone absorption is the same in WT and MR1^-/-^ mice. Levels of cefoperazone in intestinal contents were analyzed by UPLC/QTof-MS after one, three and five days of antibiotic treatment. Data shown are normalized based on the total intensity of each run and are mean ±SD. Day 1: p = 0.50, n = 2 mice per groups; Day 3: p = 0.72, n = 3 mice per group; Day 5: p = 0.70, n = 3 mice per group.

We next sought to characterize the microbiotas of MR1^-/-^ and WT mice. Fecal microbiota compositions of both WT and MR1^-/-^ mice were determined before and after antibiotic treatment. Prior to antibiotic treatment, WT mice were dominated by *Porphyromonadaceae* (~32%), while *Lachnospiraceae*, *Lactobacillaceae*, unclassified *Clostridiales*, and *Erysipelotrichaceae* comprised ~12–15% each. In contrast, the microbiota of MR1^-/-^ mice contained ~10% *Bacteroidaceae*, a family undetectable in WT mice before antibiotic treatment. *Desulfovibrionaceae* and unclassified *Burkholderiales* were also found to comprise a small portion (less than 2%) of the population of MR1^-/-^ mice and were also not seen in WT mice before antibiotics. Furthermore, MR1^-/-^ mice exhibited a greater proportion of *Porphyromonadaceae*, *Lactobacilliaceae*, and *Lachnospiraceae* compared to WT mice. Following antibiotic treatment, MR1^-/-^ mice displayed a relative abundance profile similar to their pre-treatment profile, while WT mice exhibited a loss of diversity and were dominated by *Enterococcaceae* and *Bacteroidaceae*, which comprised ~95% of the population (Fig 3A). MR1^-/-^ mice displayed significantly more richness than WT mice, both before and after antibiotic treatment (Fig 3B). Principle coordinate analysis depicts significant separation between WT and MR1^-/-^ communities regardless of treatment status, illustrating the dissimilarity of these samples. Each treatment group clustered together, demonstrating that intra-group samples were more similar to one other than to samples from any other group. Samples from WT mice treated with antibiotics did not cluster tightly together and were markedly more dissimilar to each other and to all other groups (Fig 3C). Intestinal contents were analyzed for the presence of cefoperazone during the pre-treatment period to determine if differential absorption or breakdown of the antibiotic could account for the differences in microbiota disruption and ultimately colonization resistance. Similar levels of cefoperazone were observed in WT and MR1^-/-^ mice after one, three, or five days of antibiotic treatment (Fig 4).

**Fig 5:**
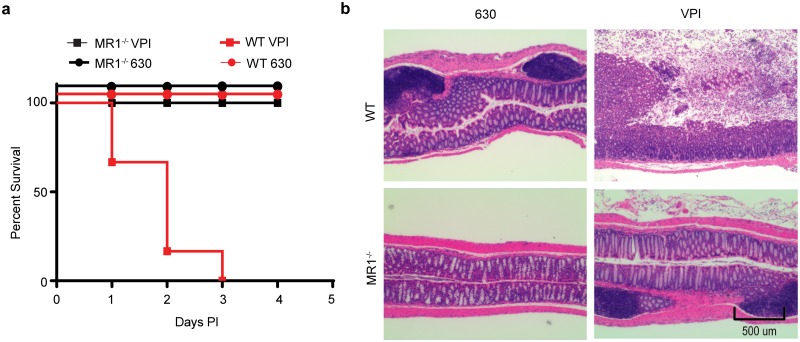
MR1^-/-^ mice are resistant to highly virulent strains of *Cd*. (A) WT and MR1^-/-^ mice were inoculated with either 1x10^3^ *Cd*630 or *Cd*VPI spores and assessed for disease daily. (B) H&E staining of colons harvested at time of euthanasia or at the study endpoint (4 days PI). n = 12 mice per group.

Infection with *Cd* strain 630 causes attenuated disease in mice, so we next sought to determine whether MR1^-/-^ mice remained resistant to a more pathogenic *Cd* strain after an extended treatment of cefoperazone (ten days) [26, 27]. WT and MR1^-/-^ mice were infected with 1x10^3^ spores of *Cd* strains exhibiting either low virulence (*Cd*630) or high virulence (VPI 10463 (*Cd*VPI) (Fig 1B). Although colonization could not be determined in WT mice infected with *Cd*VPI as these mice get sick quickly, MR1^-/-^ mice were assessed for *Cd* colonization and no bacteria were detected in these mice. In contrast to *Cd*630 (which exhibited 100% survival in both mouse strains), WT mice infected with *Cd*VPI exhibited severe disease and succumbed to infection within three days, while MR1^-/-^ mice survived the length of the experiment (Fig 5A). Histological examination of colonic tissue revealed increased inflammation, tissue damage, and cellular infiltration in WT as compared to MR1^-/-^ mice (Fig 5B). These differences were most striking in mice infected with *Cd*VPI. Although MR1^-/-^ mice did exhibit some minor inflammation, there were no detectable *Cd* bacteria isolated from these mice at any time point. In contrast, WT mice infected with *Cd*VPI showed severe disease consistent with the known virulence of this strain. Taken together, these results demonstrate that even after prolonged antibiotic pretreatment, or inoculation with a highly virulent *Cd* strain, MR1^-/-^ mice remain resistant to *Cd* colonization.

### Colonization resistance is independent of breeding location

**Fig 6:**
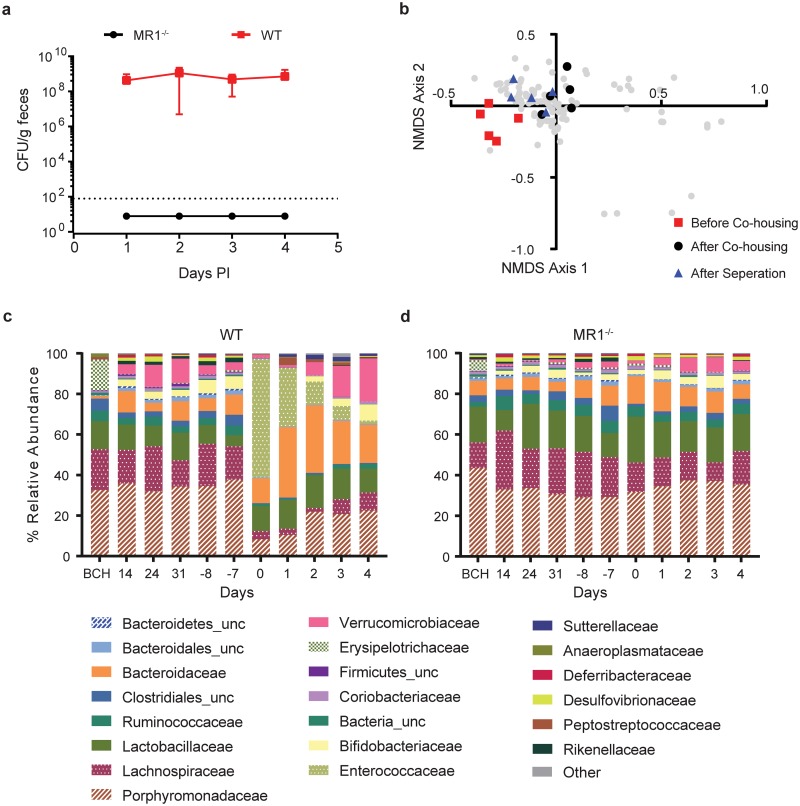
Altered *Cd* colonization resistance is not due to mouse strain breeding site differences. (A) WT and MR1^-/-^ mice were co-housed for one month, separated for three weeks, and then put through the cefaperazone model of CDI. Colonization levels were determined for four days PI. Data presented are mean ±SD. WT n = 5 MR1^-/-^ n = 6. (B) β-diversity throughout co-housing. 2-D non-metric multidimensional scaling (NMDS) plot of ThetaYC distances for all samples collected during the experiment. WT samples taken before, on the last day of co-housing, and on the last day of separation are highlighted in red, black and blue, respectively. Remaining samples are depicted gray. Relative abundance of microbiota present in WT n = 5 (C) and MR1^-/-^ n = 6 (D) mice before co-housing (BCH), during (days 14, 24, and 31), three weeks after separation (day -8), upon initiation of cefoperazone treatment (day -7), after cefoperazone treatment (day 0), and after *Cd* inoculation (days 1–4).

Vendors and breeding location can have a significant impact on microbiome study results [28, 29]. One possible explanation for the microbiota differences between WT and MR1^-/-^ mice was the location of breeding, with MR1^-/-^ mice bred in-house and WT purchased from Jackson Laboratory. To exclude this possibility, WT and MR1^-/-^ mice were co-housed for four weeks to normalize the microbiota between the two strains, separated for three weeks and then assessed for *C*. *difficile* colonization resistance using the cefoperazone pretreatment model (Fig 1C). Co-housed mice were separated prior to antibiotic treatment and infection to allow time for host-specific factors such as MAIT cells to influence the composition of the newly normalized microbiota. Co-housed WT mice were colonized to high levels while co-housed MR1^-/-^ mice continued to exhibit complete resistance to *Cd* colonization, as previously observed (Fig 6A). To further show that this phenotype was not breeding site specific, we bred WT C57Bl6 mice in the same room where the MR1^-/-^ mice were bred for at least five generations. These in-house mice were put through the standard CDI model described previously and exhibited similar colonization levels to purchased WT mice (data not shown). We believe the fact that WT mice co-housed with MR1^-/-^ mice or WT bred in the same facility and room with the MR1^-/-^ mice are both colonized by *Cd* at high levels supports our conclusion that differences seen between these mouse strains are not simply due to original breeding location.

Microbiota composition was monitored throughout co-housing and infection. There was a shift in the microbiota of WT mice during co-housing with MR1^-/-^ mice (Fig 6B). If the presence of MR1+ cells had no influence on the microbiota, and breeding location was the sole reason for the difference in microbiota, we expected to see an initial shift during the co-housing period, followed by stable colonization of the WT mice with bacteria associated with the MR1^-/-^ mice and the FDA vivarium. However, three weeks after co-housing the microbiome of the WT mice began to revert towards the original state but did not fully recover (Fig 6B). The microbiota of MR1^-/-^ mice did not exhibit substantial changes throughout the experiment. Relative abundances of the microbiota in both strains exhibited the same trend (Fig 6C and 6D). Together, these data show that differences in microbiota composition of WT and MR1^-/-^ mice are not a result of breeding location.

### Colonization resistance is conferred by the microbiota

**Fig 7:**
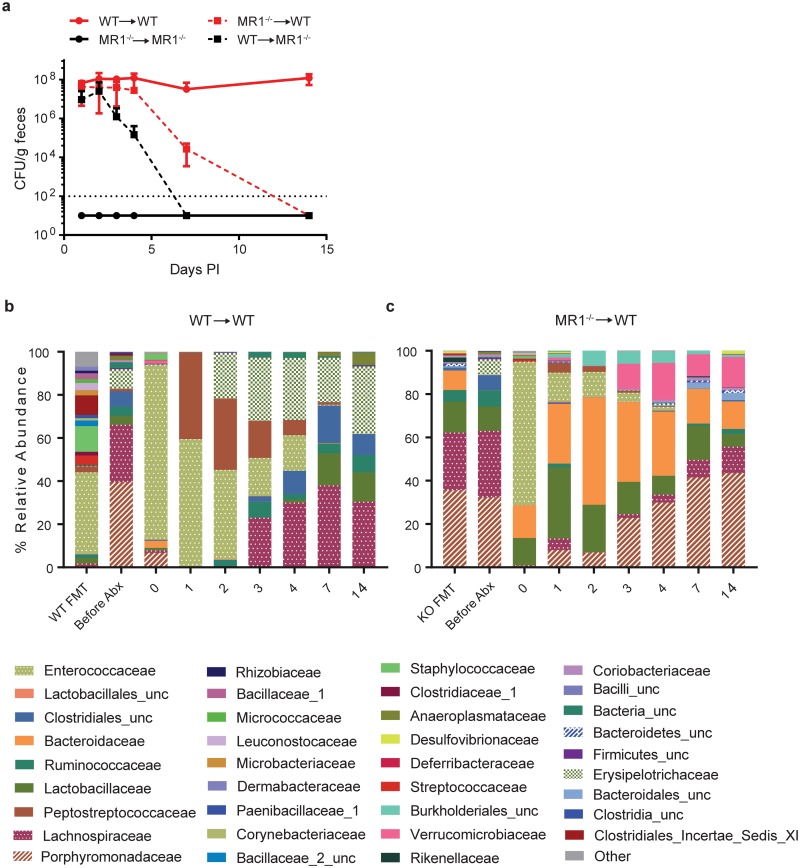
*Cd* colonization resistance is transferrable to susceptible mice. (A) WT and MR1^-/-^ mice were treated as pictured in Fig 1d and then taken off cefoperazone one day prior to FMT administration. Each strain was divided into two groups and inoculated with antibiotic treated stool from either autologous stool, or stool from the opposite strain. Colonization levels were tracked over time (B & C). Assessment of microbiota composition over time following FMTMR1^-/-^ → MR1^-/-^ n = 3, WT→ MR1^-/-^ n = 3, WT→WT n = 4 MR1^-/-^ →WT n = 3. Data presented are mean ±SD of one experiment.

To determine if the microbiota composition of MR1^-/-^ mice was responsible for the colonization resistance phenotype, FMT experiments were performed. WT or MR1^-/-^ SPF mice were given cefoperazone, then transplanted with either autologous stool, or stool from the opposite mouse strain (Fig 1D). As expected, MR1^-/-^ mice that received MR1^-/-^ stool did not display any colonization above the level of detection (Fig 7A, solid black line) and WT mice that received WT stool exhibited high levels of colonization (Fig 7A, red solid line). MR1^-/-^ mice that received stool from WT mice were initially colonized, however only transiently, dropping to 10^4^ CFU/g by day four and were undetectable by day seven (Fig 7A, dashed black line), indicating that the transferred WT microbiota did not allow persistent *Cd* colonization. Importantly, susceptible WT mice that received MR1^-/-^ donor stool were initially colonized at high levels, but *Cd* was cleared rapidly, and no *Cd* were detectable in stool at day 14 (Fig 7A, dashed red line). The composition of the microbiome in WT mouse recipients was tracked over the course of the experiment (Fig 7B and 7C). After FMT and *Cd* inoculation, WT mice given FMT from MR1^-/-^ donors exhibited higher levels of *Bacteroidaceae*, *Porphromonadaceae* and *Verrucomicrobiaceae* in their stool when compared to those given WT FMT. In contrast, mice that received autologous stool exhibited higher levels of *Enterococcaceae* and *Lachnospiraceae*. These results suggest that the observed MR1^-/-^ colonization resistance is controlled by members of the altered microbiota in these mice.

**Fig 8:**
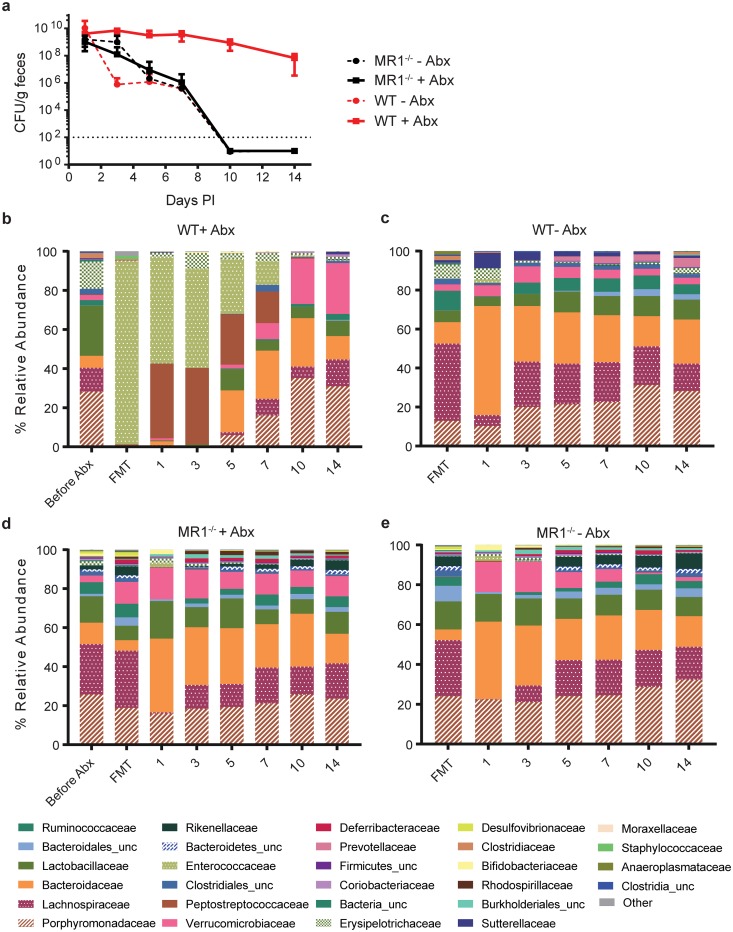
Microbiota of MR1^-/-^ mice is responsible for colonization resistance. (A) Germ-free C57BL/6NTac mice received FMT from SPF mice. Each donor (SPF WT and MR1^-/-^) strain was separated into two groups, with and without antibiotic treatment. Colonization levels for each group were determined up to 14 days PI. (B-E) Microbial composition of previously germ-free mice following FMT. Data shown are mean ±SD of 2 independent experiments. n = 10 mice per group.

We next sought to confirm this microbiota associated resistance phenotype from MR1^-/-^ mice using germ-free mice as FMT recipients [30]. WT germ-free mice received FMT derived from stool collected from conventional WT or MR1^-/-^ donors either with or without antibiotic pretreatment. Fecal transplants were performed one day after the cessation of antibiotics (if treated), and one day prior to inoculation with *Cd* spores (Fig 1E). Initially all groups exhibited similar levels of colonization, however only mice that received FMT from WT antibiotic-treated mice remained colonized throughout the experiment, with all other groups clearing detectable *Cd* by day 10 (Fig 8A). As predicted from relevant literature and experiments presented here, the control (non-antibiotic treated) mice were protected from *Cd* colonization, while germ-free mice given FMT from antibiotic treated WT mice became colonized [21]. Importantly, MR1^-/-^ stool was protective, even following antibiotic treatment. These data indicate that the *Cd* colonization resistance observed in MR1^-/-^ mice is provided by the microbiome as the resistance phenotype can be transferred to susceptible germ-free mice. 16S rRNA gene sequencing analysis of the WT antibiotic-treated mouse derived FMT material and samples isolated immediately after FMT administration showed relatively low diversity and a microbiota dominated by *Enterococcaceae* in the days immediately post-infection (Fig 8B). Minimal variation in relative abundance was observed among the microbiota of the three remaining groups, those that cleared CDI within 10 days post-infection. As in previous experiments, *Porphyromonadaceae*, *Lachnospiraceae*, *Lactobacillaceae* and *Bacteroidaceae* made up ~60–80% of each sample (Fig 8C–8E).

**Fig 9:**
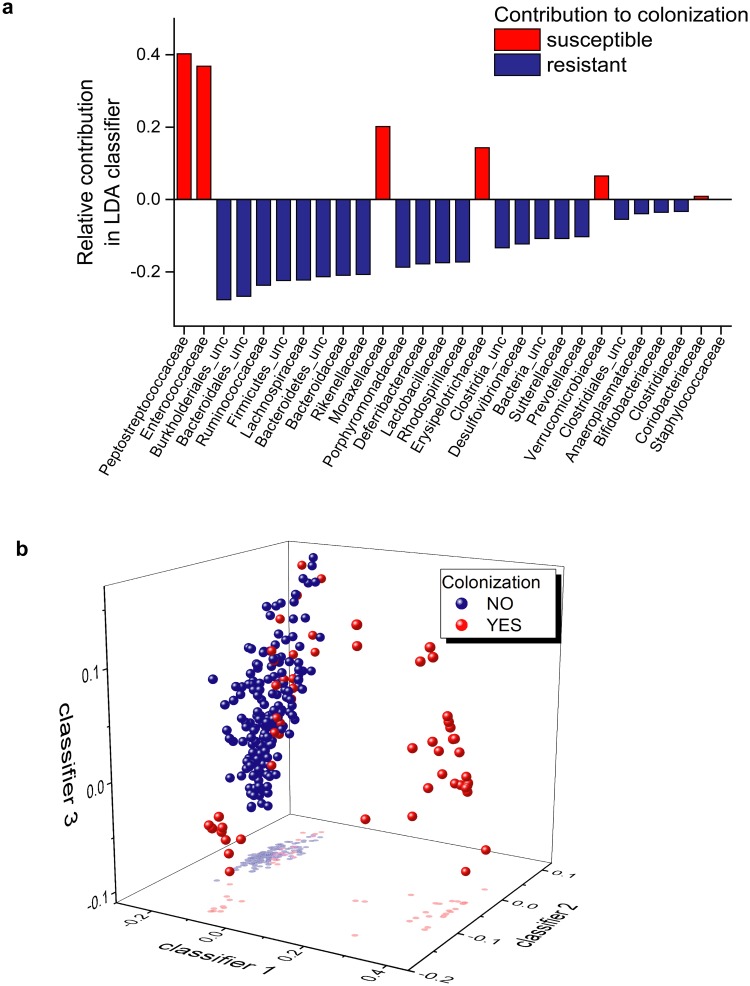
RLDA identifies families that contribute to colonization susceptibility. (A) Regularized Fisher’s linear discriminant analysis (RLDA) was performed using the relative abundance data collected from germ-free mice administered FMT from WT and MR1^-/-^ SPF mice and colonization status as in Fig 5. The relative contribution of bacterial colonies differentiates the samples that undergo colonization and samples that do not. Bacteria are ordered in descending order of importance. (B) 3-D plot depicts RLDA of colonization status of previously germ-free mice. Colonized samples (red dots) vs non-colonized samples (blue dots) are arranged based on differentiator vectors. Samples are colored based on final colonization status, no stratification was performed based on timepoint.

Regularized Fisher’s linear discriminant analysis (RLDA) classification was performed to detect bacterial populations that serve as main discrimination markers between the group that undergoes colonization versus the group that does not. Ninety percent of differentiation in colonization pattern was explained by 14 families. *Peptostreptococcaceae*, *Enterococcaceae*, and *Moraxellaceae* were associated more with colonization, while *Burkholderiales*, *Bacteroidales*, *Ruminococcaceae*, *Firmicutes*, *Lachnospiraceae*, *Bacteroidetes*, *Bacteroidaceae*, *Rikenellaceae*, *Porphyromonadaceae*, *Deferribacteraceae* and *Lactobacillaceae* were predicted to play a role in resisting colonization (Fig 9A). Fig 9 demonstrates the relative contribution of these families to colonization. Notably, *Peptostreptococcaceae*, the family to which *Cd* belongs, was the highest contributor, validating this method. Additional visual representations of RLDA demonstrate clusters of the samples under a new coordinate system. From this perspective, the presence of the abovementioned bacterial populations clearly separates two groups and as such can serve as a marker for potential colonization (Fig 9B). The classification and prediction of the RLDA support our conclusion that microbiota of MR1^-/-^ mice are responsible for the *Cd* colonization resistance phenotype.

## Discussion

Here we have shown that MR1^-/-^ mice exhibit resistance to CDI. This resistance can be transferred to susceptible mice via FMT, suggesting that MR1^-/-^ mice have an altered microbiota structure, which may be due to the absence of MAIT cells and other MR1 restricted T cells. The minimal changes in the microbiota of MR1^-/-^ mice following antibiotic treatment may be responsible for the inability of *Cd* to colonize. However, increased colonization resistance was observed independent of antibiotic pre-treatment (cefoperazone, streptomycin, five-antibiotic cocktail) or *Cd* strain (Cd630 or CdVPI) used.

Colonization resistance was transferrable from MR1^-/-^ mice to susceptible germ-free or antibiotic treated SPF WT mice. While colonization was observed in MR1^-/-^ mice directly after FMT, detectable *Cd* quickly cleared. This transient colonization is likely associated with the timing of infection relative to FMT administration, which may have been insufficient to allow the FMT administered microbiota to establish in the new environment. However, we believe this model more accurately depicts clinical usage of FMT against recurrent CDI. In susceptible WT mice, once the resistant microbiota (from MR1^-/-^, or untreated WT) had time to engraft, colonization levels declined. However, it appears that the initial MR1^-/-^ related microbiota outcompeted the incoming WT FMT material, leading to *Cd* clearance. Others have observed a similar delay in *Cd* clearance following FMT administration in a mouse model of recurrent disease, showing that an “establishment” period may be necessary for newly transferred microbiota to provide colonization resistance [26].

While it is accepted that the microbiota provides colonization resistance against CDI, the specific organisms, or mechanisms, that mediate colonization resistance remain to be elucidated [17, 19, 31]. The increased abundance of *Porphyromonadaceae*, *Lactobacilliaceae*, and *Lachnospiraceae* in MR1^-/-^ mice after antibiotic treatment as compared to WT mice suggest that these families may play a role in *Cd* colonization resistance. This is consistent with a study that found *Lachnospiraceae*, *Porphyromonas*, *Lactobacillus*, and *Alistipes* correlated with colonization resistance [24]. Additionally, pre-colonizing with *Lachnospiraceae* was reported to significantly reduce *Cd* colonization and toxin levels [32]. Another study has shown a role for *C*. *scindens* in the reduction of *Cd* colonization [33]. Our studies and analyses predict roles for *Porphyromonadaceae* and *Lachnospiraceae*, as families that may contribute to colonization resistance, and *Enterococcaceae* which may indicate susceptibility; this is important as these could be developed into biomarkers of FMT efficacy.

The interactions between the immune system and the microbiota have been reviewed from the perspectives of the microbiota controlling the immune system and the immune system controlling the microbiota [34]. However, many of the studies involving the latter have focused on an immune defect leading to disrupted microbiota and associated disease. The data presented here describe a previously unappreciated phenotype in which the presence of MR1 is associated with decreased gut microbial diversity and susceptibility to *Cd* colonization. Studies of CD44, PD-1 and IL1-R2 describe a similar phenomenon, whereby the microbiota associated with these knockout mice promoted reduced disease symptoms. In an induced model of experimental autoimmune encephalomyelitis (EAE), FMT from CD44 KO mice into WT mice ameliorated disease symptoms. WT mice that received the CD44 KO FMT also exhibited increased Simpson diversity, Tregs, and short chain fatty acids [35]. In a mouse model of inflammatory bowel disease, PD-1 deficient mice administered dextran sodium sulfate (DSS) to induce colitis exhibited less susceptibility to disease, including decreased weight loss and significantly lower histology scores as compared to their WT counterparts. However, co-housing of PD-1 KO mice with WT mice resulted in the transfer of disease susceptibility to KO from WT mice and not resistance from KO to WT as observed in our study [36]. A similar model of DSS induced colitis investigated WT and IL1-R2 KO mice in which the KO mice exhibited less colon shortening, a clinical sign of disease. As in the PD-1 study, co-housing of IL1-R2 KO mice and WT mice suppressed the disease resistance in the KO mice [37].

A recent study has shown that MR1^-/-^ non-obese diabetic (NOD) mice exhibited significantly greater intestinal permeability than their MR1^+/-^ NOD littermates, defective function of ileum epithelial cells, and increased infiltration of lymphoid cells into the lamina propria [38]. Although the mechanisms by which MR1 promotes intestinal homeostasis remain undefined, these defects likely have a significant impact on the constituents of the gut microbiota. Importantly, the Rouxel et al. study did not examine whether their MR1-deficient strain exhibited microbiota dysbiosis, as we have found here. The increased intestinal permeability observed in MR1^-/-^ mice could lead to greater tissue infiltration of constituents of the microbiota and their products, including riboflavin metabolites, resulting in activation of MAIT cells and other MR1-restricted T cells. The resultant production of pro-inflammatory cytokines could have several downstream consequences, including altered APC responses and modification of the microbiota. The work presented here describes a unique role for MR1 in modulation of the microbiota and susceptibility to *C*. *difficile* infection. Further study of the immune mechanisms that influence the microbiota may open the door to a better understanding of the factors that influence microbiota diversity and disease susceptibility.

## Conclusions

MR1^-/-^ mice exhibit altered microbiota composition, which results in *Cd* colonization resistance even following antibiotic treatment. Although high levels of cefoperazone were detected in the gut, the microbiota of these mice appears to be resistant to antibiotic killing. The resistance phenotype is transferred by giving FMT, suggesting that the microbiota associated with MR1^-/-^ mice is responsible for their observed *Cd* colonization resistance, even after antibiotic treatment. Future studies of the microbiome in MR1^-/-^ mice will lead to a greater understanding on the mechanisms of *Cd* colonization resistance and the identification of biomarkers of FMT efficacy against this pathogen.

## Methods

### Animals

All animal experiments were approved by the Food and Drug Administration (FDA) institutional animal care and use committee (IACUC) guidelines.

### Specific pathogen free mice (SPF)

Adult wild-type C57BL/6J mice were obtained from The Jackson Laboratory (Bar Harbor, ME), while MR1^-/-^ mice were originally generated by Treiner et al. [6]. The MR1^-/-^ mice were obtained from Ted Hansen (Washington University in St. Louis, St. Louis, MO) and bred at CBER/FDA. MR1^-/-^ mice are genotyped by TransnetYX, Inc. (Cordova, TN) using the following primers: 5’ CTTTCCTGAGCCGCTCGAA 3’ and 5’CCAGCTCCAAAATGCAGCC 3’. The MR1^-/-^ mice were originally generated on a mixed B6/129OlaHsd background. The mice were back-crossed to the C57BL/6N background (10 generations) and then the B6J background (10 generations).

### Germ-free mice

Germ-free C67BL/6Tac mice were purchased from Taconic and housed in flexible film positive-pressure isolators (Class Biologically Clean, Ltd., Madison, WI) until immediately prior to fecal microbiota transplantation (FMT). To ensure mice remained sterile until FMT administration, mice and the isolator environment were sampled upon arrival at the facility and weekly thereafter. After administration of FMT, the mice were housed under standard specific pathogen free (SPF) BSL-II conditions.

### Mouse co-housing

Two wild-type C57Bl/6J mice were co-housed with two MR1^-/-^ mice for a total of four mice per cage. The mice were co-housed for four weeks and then separated and re-housed with only mice of the same strain. Mice remained in single-strain housing for the remainder of the experiment. Three weeks after separation, all mice began the standard cefoperazone pretreatment described below.

### *Clostridium difficile* strains

*Clostridium difficile* 630 (ATCC BAA-1382) and Virginia Polytechnic Institute (VPI) 10463 (originally isolated from an abdominal wound) strains were stored as spores in sterile water at 4°C. An initial stock of *Cd*630 spores was made and all experiments were performed using this stock. Briefly, five *Cd*630 cultures were grown overnight at 29°C in 5 mL Columbia broth. The cultures were then added to 45 mL of Clospore and allowed to grow at 37°C for approximately 10 days. Spores were centrifuged at 1300 x g for 10 min and washed in sterile water 3x before being combined and resuspended in sterile water and transferred to a 2 mL tube (Corning 430909, Corning, NY) for long-term storage. *Cd* strains were grown and plated in an anaerobic chamber (Coy Industries, Grass Lake, MI).

### *Clostridium difficile* inoculation preparations

Spore stocks were enumerated one day prior to each *in vivo* infection. An aliquot of spores was diluted 1:10 in sterile water and heat-treated for 30 min in a 65°C water bath after which the spores were serially diluted and plated on Brain Heart Infusion agar supplemented with 0.5% yeast, 0.1% cysteine (BHIS), and 0.1% taurocholate (BHIS+Tc). Plates were incubated in anaerobic conditions at 37°C overnight and CFU counted to determine the concentration of the spore stock. All animal experiments were performed with *Cd*630 spores unless otherwise noted.

Spores stocks were diluted to 1x10^4^ spores/mL in sterile water based on the enumeration from the previous day. The preparation was then heat-treated for at least 30 min at 65°C to kill any vegetative *Cd* cells present in the preparation. Mice were inoculated by oral gavage with 100 μL (1x10^3^ CFU) heat-treated spores. Immediately after infection, an aliquot of the spore inoculum was plated on a BHIS+Tc agar plates to determine the actual number of spores administered to each mouse.

For vegetative cell *in vivo* infection, *Cd*630 spores were streaked for isolation onto a BHIS+Tc agar plate to produce vegetative *Cd* colonies. One night prior to infection, single colonies were inoculated into BHIS broth and grown ON at 29°C. The morning of infection, cultures were back diluted 1:5 in BHIS and incubated at 37°C until OD_600_ was approximately 1 (~two hours). Cells were centrifuged, washed three times with anaerobic PBS and combined in a single tube. OD_600_ was measured again to quantify the cells using the following equation: y = 4x10^7^(OD_600_)– 6x10^6^. *Cd* cells were diluted to the appropriate concentration (5x10^6^ cells/mL) in anaerobic PBS. Syringes were loaded under anaerobic conditions before being transported to the CBER vivarium for inoculation. Mice were administered 1x10^6^ vegetative cells in 200 μL by oral gavage. The vegetative *Cd* cell suspension used to infect the mice was immediately plated after infection in order to determine the actual number of *Cd* cells given to each mouse.

### Murine models of *Clostridium difficile* infection

Adult C57Bl/6J mice were administered antibiotics *ad libitum* in sterile drinking water for five days which was refreshed every other day. Cefoperazone 0.5 mg/mL; MP Biomedicals, Santa Ana, California) is the considered the standard model of antibiotic pretreatment in these studies and is used in all experiments unless otherwise noted [22]. Alternative antibiotic pretreatment strategies include the administration of streptomycin sulfate (5mg/mL; VWR, Radnor, PA) [24] and a cocktail comprised of five antibiotics: kanamycin (0.4 mg/mL), gentamycin (0.035 mg/ml), colistin (850 U/mL, metronidazole (0.0215 mg/mL), vancomycin (0.045 mg/mL) adapted from Chen et al. [21]. All antibiotics for the 5-antibiotic cocktail were purchased from Sigma-Aldrich (St. Louis, MO). Two days prior to *C*. *difficile* infection, antibiotic treatment was discontinued, and the mice were given normal drinking water for the remainder of the experiment. When the 5-antibiotic cocktail was administered, a single dose of clindamycin (10mg/kg) was injected IP one day prior to infection. *Cd* was administered by oral gavage as described above. The doses used to infect the mice were chosen as they have been previously shown to sufficiently cause *Cd* colonization [26, 39].

Fecal pellets were collected throughout the duration of the experiments and either immediately diluted in anaerobic PBS and plated to assess bacterial colonization, or immediately stored at -80°C for sequencing analysis. After plating, residual fecal samples were stored at -80°C for subsequent sequencing analysis.

### Survival study

One survival experiment was conducted as using SPF WT and MR1^-/-^ mice inoculated with *C*. *difficile* strains 630 and VPI 10463. The experiment included four groups, each with four mice per group. All mice inoculated with VPI were monitored closely (at least twice daily) for symptoms of severe CDI. No additional animal welfare considerations were taken, and no special training was needed to monitor the mice. Any mice observed to be hunched, unkempt/scruffy or experienced a documented weight loss of 20% of their pre-inoculation body weight was considered to have reached their humane endpoint and were euthanized immediately. One of the four WT mice inoculated with VPI succumbed to infection overnight (between the evening and early morning monitoring) and the carcass was removed from the cage as soon as it was discovered in the morning. The other three mice in that group reached their humane endpoints by day 3 PI and were euthanized. All mice in the other three groups survived to day 4 (the study endpoint) when the mice were euthanized, and tissues harvested for histopathologic examination.

### Assessment of bacterial colonization

*C*. *difficile* colonization was assessed under anaerobic conditions by plating stool (serially diluted in anaerobic PBS) on BHIS agar with tauracholate (1mg/mL), cefoxitin (8 μg/mL), and D-cycloserine (250 μg/mL). Plates were incubated overnight at 37°C before counting CFUs. All colonization level data are shown as mean ±SD.

### Fecal microbiota transplantation

Fecal microbiota transplantation (FMT) material was prepared under anaerobic conditions. One fecal pellet was collected directly into anaerobic PBS at a ratio of approximately one pellet to 150 μL PBS for each animal to be inoculated. Stool was homogenized and centrifuged to pellet debris. FMT material is loaded into syringes in the anaerobic chamber and transported to the CBER vivarium where each mouse was administered 100 μL FMT material by oral gavage. FMT was performed one day prior to *Cd* inoculation.

### Histopathology

Cecum and colon tissue were harvested and immediately fixed in 10% neutral buffered formalin for 24–48 hours. Tissues were then transferred to freshly prepared 70% ethanol for storage. Tissues were paraffin embedded, sectioned, and stained with hematoxylin and eosin (H&E) by Histoserv, Inc. (Gaithersburg, MD). Microscopy was performed on the Olympus CKX53 Inverted microscope (Center Valley, PA) and pictures were captured with an INFINITY 1 color camera with INFINITY ANALYZE software 6.5.2 (Luminera, Ottawa, ON).

### Open metabolic profiling by UPLC/QTof-MS

Mice were treated with cefoperazone 0.5 mg/mL for one, three, or five days before the contents from the small intestines were collected, immediately snap frozen, and stored at -80°C for future analysis.

To prepare samples for LC/MS analysis, intestinal contents (~250 mg) were mixed with extraction solvent (1:1 MeOH: water) at a ratio of 1:5 (w/v). The mixture was vortexed for 2 min, followed by 10 min sonication. After centrifugation at 13,000 rpm for 15 min at 4 °C, the supernatant was then transferred to autosampler vials for analysis.

A 5 μL aliquot of the extracted supernatant was introduced into a Waters Acquity Ultra Performance Liquid Chromatography (UPLC) system (Waters, Milford, MA) equipped with a Waters bridged ethyl hybrid (BEH) C8 column with a dimension of 2.1 mm × 10 cm and 1.7 μm particle size. The column was held at 40°C. The UPLC mobile phase consisted of 0.1% formic acid in water (solution A) and 0.1% formic acid in acetonitrile (solution B). While maintaining a constant flow rate of 0.4 mL/min, the plasma metabolites were eluted using linear gradients of 2–80% solution B from 0 to 15 min, and 80–98% solution B from 15 to 17 min. The final gradient composition was held constant for 2 min, followed by a return to 2% solution B at 19.1 min. Mass spectrometric data were collected with a Waters QTof Premier mass spectrometer (Waters, Milford, MA) operated in positive and negative ionization electrospray modes, as reported previously. Briefly, MS^E^ analysis was performed on a QTof mass spectrometer set up with 5 eV for low collision energy and a ramp collision energy ranging from 20 to 30 eV. Full scan mode from *m*/*z* 80 to 1000, and from 0 to 22 min was used for data collection. The cefoperazone intensity was obtained using Micromass MarkerLynx XS Application Version 4.1 (Waters, Milford, MA) with extended statistical tools and normalized to the total intensity of each run.

## 16S rRNA gene sequencing

DNA extraction and 16s rRNA gene sequencing were performed by the Microbial Systems Molecular Biology Laboratory (MSMBL) core at the University of Michigan (Ann Arbor, MI) as previously described [26]. Briefly, DNA was extracted from the stool samples using the Qiagen PowerMag 96-well kit optimized for the epMotion 5075 TMX (Eppendorf, Hauppauge, NY). Amplicon sequencing was achieved by amplifying the v4 region of the 16s rRNA gene using a set of barcoded dual-index primers developed by Kozich et.al [40]. Each primer includes the appropriate Illumina adapter, an 8-nt index sequence, a 10-nt pad sequence, a 2-nt linker, and the v4 specific primer (Read1: TATGGTAATTGTGTGCCAGCMGCCGCGGTAA and Read2: AGTCAGTCAGCCGGACTACHVGGGTWTCTAAT). Sequencing was performed with MiSeq v2 2x500 chemistry on the Illumina MiSeq platform according to manufacturer’s specifications with modifications for the custom primer set.

### Data analysis

Data was analyzed using High-performance Integrated Virtual Environment (HIVE) computational platform designed for big data regulatory research of biomedical data [41].

16S rRNA gene sequencing analysis was performed using the mothur (v.1.39.5) analysis pipeline integrated into the HIVE platform. Analysis was performed according to the mothur MiSeq SOP [40, 42]. Sequences were classified using a naïve Bayesian classifier with the Ribosomal Database Project (RDP) training set (version 16) at an 80% bootstrap confidence score cutoff and aligned to the SILVA bacterial reference database. Chimeric sequences were identified using the VSEARCH algorithm and removed from the dataset [43]. A mock community (HMP or Zymo) was simultaneously sequenced with the fecal samples to assess sequencing error rates. The error rates associated with each experiment are as follows: before and after antibiotics (0.029%), co-housing (0.023%), FMT (1.265) and germ-free (0.029%). To avoid uneven sampling bias, subsampling was performed to normalize the samples in each experiment. The subsampling strategy was based on the lowest reasonable number of sequences per sample with 1000 sequences being the minimum threshold. The before and after antibiotics experiment, co-housing, FMT and germ-free experiments were rarefied to 2000, 8000, 1000, and 1800 sequences per sample respectively.

Data were classified based on phylotypes at the family level. Relative abundance graphs are depicted as % mean relative abundance. “_unc” denotes sequences unclassified at the family level; the lowest level identified is listed. Only taxa >1% is displayed, any taxa under the threshold was combined as “Other”. α-diversity was assessed with Chao1 richness estimations. Data presented in relative abundance graphs and richness estimations are mean ±SD. Comparison of phylotypes was performed by calculating dissimilarity matrices based on the metric by Yue and Clayton (ThetaYC) [44]. ThetaYC distances are visualized by principle coordinate analysis (PCoA) or 2-D non-metric multidimensional scaling (NMDS) plots.

Relative abundance data was also subjected to a classification procedure known as regularized form of Fisher’s linear discriminant analysis (RLDA) as implemented in HIVE platform. Each sample is represented in the form of a vector in a multidimensional space of its measurements that in this case are the abundances of bacterial species based on ribosomal 16SrRNA sequencing. RLDA requires known categorization schema for samples (colonized vs non-colonized) in order to determine the discriminant vectors best differentiating the groups from each other. It performs linear transformation of the coordinate system in such a way that maximizes in-between group variability and minimizes within group variability. As an outcome, it produces a linear combination of original measurements and sorts the bacterial abundances in order of their contribution into differentiation. Bacterial communities most responsible for separation between groups are detected. While being similar to a dimensionality reduction procedure like principal component analysis (PCA), it has a significant difference in that the PCA maximizes overall variability of the data regardless of grouping of the samples, and RLDA maximizes variability between sample groups. RLDA also produces a visualization in form of clusters of samples after translation of the coordinate system by multiplying the original measurement matrix to the matrix of eigenvectors. This representation creates 3-D pictures demonstrating separation between groups in this new coordinate system. Sequence data are deposited in the NCBI SRA database accession # PRJNA493800.

### Statistical analysis

Unless otherwise noted, statistical analysis was performed using GraphPad Prism 7.01 (GraphPad Software, La Jolla, CA). To assess differences between richness (Chao1) in WT and MR1^-/-^ mice before and after antibiotics (Fig 3B), one-way ANOVA was performed with multiple comparisons and Benjamani, Krieger and Yekutieli method to control the False Discovery Rate. A t-test with the two-stage step-up method of Benjamani, Krieger and Yekutieli was performed to identify differences in cefoperazone intensity from intestinal contents. P value <0.05 was considered significant.
